# No difference in blood loss between posterior-cruciate-ligament-retaining and posterior-cruciate-ligament-stabilized total knee arthroplasties

**DOI:** 10.1007/s00167-013-2818-z

**Published:** 2014-01-03

**Authors:** Deniz Cankaya, Bulent Ozkurt, Cemal Aydin, A. Yalcin Tabak

**Affiliations:** Department of Orthopaedic and Traumatology, Ankara Numune Training and Research Hospital, 06100 Altindag, Ankara Turkey

**Keywords:** Total knee arthroplasty, Posterior cruciate ligament substituting, Drainage, Blood loss

## Abstract

**Purpose:**

Posterior-cruciate-ligament-retaining (PCR) and posterior-cruciate-ligament-stabilized (PS) arthroplasties are two major common practices in total knee arthroplasty (TKA). The hypothesis of the present study was that compared with the PCR technique, the PS technique is associated with a higher amount of postoperative blood loss and greater need for blood transfusion in cemented TKA.

**Methods:**

In this prospective, randomized study, 100 patients diagnosed with primary knee osteoarthritis were randomly assigned to either the PCR group (Group I) or the PS group (Group II). The exclusion criteria were rheumatological joint disease, previous knee surgery, anticoagulant therapy and hypertension. There were no significant differences in age, body mass index and gender, between the groups. The haemoglobin and haematocrit levels of each patient were recorded preoperatively and on postoperative days 1, 3 and 5. The postoperative suction drainage and blood transfusion volumes were also recorded.

**Results:**

There were no statistically significant differences in haemoglobin or haematocrit levels between the groups on postoperative days 1, 3 and 5. There were also no statistically significant differences in the total measured blood loss volume, postoperative drainage amounts or transfusion rates between the groups.

**Conclusion:**

Use of the PS technique during cemented TKA does not appear to influence the amount of perioperative blood loss or the need for postoperative blood transfusion in general. The clinical relevance of this study is that the difference in blood loss between the PCR and PS techniques does not need to be considered by surgeons when performing TKA.

**Level of evidence:**

I.

## Introduction

Patients undergoing total knee arthroplasty may have significantly low blood values and a high amount of blood loss after the surgical procedure. The amount of blood loss reported in the literature varies in this group of patients according to the surgical technique, clamping of the drainage site and pharmacological agent application; reported blood loss amounts include 1,100–3,030, 586–930 and 888–1,450 ml [7, 11, 18].

In total knee arthroplasty (TKA), the posterior-cruciate-ligament-retaining (PCR) and posterior-cruciate-ligament-stabilized (PS) techniques are widely used depending on the individual preference of the surgeon. Comparative analysis of these two techniques is a major topic of investigation in arthroplasty research. Regardless of the technique, approach or implant, the main source of ongoing blood loss after TKA is the cutting of cancellous bone during the surgical procedure [7, 8]. With the PS technique, there is additional cancellous bone cutting; thus, it has been suggested that there is extra blood loss from the venous sinuses of the cancellous bone in this technique. If this extra blood loss is significant, it may be a disadvantage of the PS technique that influences the surgeon’s preferences in favour of the PCR technique. To the best of our knowledge, no prospective randomized study has compared the blood loss between the PS and PCR techniques after TKA. The hypothesis of the present study was that compared with the PCR technique, the PS technique is associated with a higher amount of postoperative blood loss and greater need for blood transfusion in cemented TKA.

## Materials and methods

This study included 100 patients with knee osteoarthrosis who underwent primary total knee replacement between 2010 and 2012 according to the inclusion and exclusion criteria described below. Patients were randomized before the operation by generating random numbers with Microsoft Excel 2007 (Microsoft Corporation, Seattle, WA, USA). The PCR technique was applied to Group I (*n* = 50) and the PS technique to Group II (*n* = 50). The inclusion criteria were unilateral primary knee osteoarthritis and a patient age of 50–80 years. The exclusion criteria were rheumatological joint diseases, previous knee surgery, anticoagulant therapy, metabolic bone disease and hypertension. None of the patients had any chronic hepatic or haematological disease, malignancy history, vascular disorders or cardiac operation history. During the PCR and PS operations, a Vanguard^®^ Complete Knee System prosthesis (Biomet Inc., Warsaw, IN, USA) was implanted using the same surgical technique in all patients. Both the tibial and femoral components were implanted with bone cement. Patellar surface arthroplasty was not performed in any case. The age, sex and body mass index distributions of the groups are shown in Table 1. Considering the anthropometric and demographic data, there were no significant differences between the groups.

**Table 1:** Demographic data of the patients

	PCL retaining group	PCL stabilized group	*P* value
Gender (*n*)			n.s.
Female	42	40	
Male	8	10	
Age	66 ± 7.5	67 ± 7.1	n.s.
Height (cm)	161 ± 5.3	163 ± 6.4	n.s.
Weight (kg)	79 ± 11.9	81 ± 9.4	n.s.
Body mass index (kg/m^2^)	30.2 ± 3.8	30.4 ± 3.6	n.s
Transfusion (packed red cells/patient)	1.1 ± 0.8	1.2 ± 0.9	n.s.

The values are given as mean and standard deviation

A low dose of low-molecular-weight heparin was administered to all patients 12 h before surgery. All patients were normotensive during the perioperative period. A tourniquet was inflated to pressure of 300 mmHg after spinal anaesthesia. All operations were performed with the same surgical and spinal anaesthetic technique. A straight, longitudinal midline skin incision and medial parapatellar arthrotomy were performed. Standard soft-tissue releases and bone removals were performed to obtain balance of the ligaments. While cutting the posterior cruciate ligament, no additional measures were taken to decrease bleeding such as cauterization of the vasculature around the ligament. The intramedullary guiding hole inside the femur was closed by impaction of an autologous structural bone graft into the entry point in all patients. Both femoral and tibial prostheses were implanted with pressured bone cement. A suction drain was placed inside the knee capsule; the overlying layers were closed in the anatomical planes. Following deflation of the tourniquet, a Jones bandage was applied and the suction drain was activated. The total time of tourniquet application was recorded. Forty-eight hours after surgery, the Jones bandage and suction drain were removed. The patients were mobilized, and continuous passive motion was started just after removal of the drains. No iron or erythropoietin was given before or after surgery. No strategies to reduce blood loss, such as cell savers, bipolar sealers or pharmacological agents, were used in any patient. Blood transfusion was performed in patients with a haemoglobin value of <8.5 g/dl or with a compromised clinical condition as evidenced by tachycardia, hypotension or symptoms of anaemia during and after surgery in consultation with the anaesthesia team. All blood transfusions were applied within 2 days postoperatively. The haemoglobin and haematocrit values were recorded at postoperative 12 h and days 1, 3 and 5. Postoperative blood drainage was recorded by pouring blood from the suction drain into a measuring jar. The external scale of measuring jar was read by the same two researchers for all patients. The total blood transfusion volume was also recorded for all patients. Blood loss was calculated with the formula defined by Gross [4]. In all patients, perioperative antibiotic prophylaxis using a first-generation cephalosporin was administered to prevent infection, and the same analgesic treatment to reduce pain was administered using patient-controlled analgesia.

Low-molecular-weight heparin was administered for 4 weeks postoperatively to prevent deep vein thrombosis. A daily treatment of 40 mg enoxaparin sodium (Clexane; Aventis Intercontinental, France) was administered subcutaneously to all patients. Dynamic compression socks were applied to both lower extremities on postoperative day 2 in addition to the prophylactic enoxaparin treatment.

The study was approved by the ethics committee of Ankara Numune Training and Research Hospital with the ID number of E-13-021.

### Statistical analysis

Sample size was estimated using the amount of drainage as a primary effect variable. We assumed a difference in means of 100 ml and standard deviation of 150 ml for each group. The group sample sizes of 49 and 49 achieved a power of 0.90 to detect a difference of 100 ml between the two groups with estimated group standard deviations of 150 for each group and with a significance level (alpha) of 0.05 using a two-sided, two-sample test. Thus, the study included 50 patients in each group. All data were calculated as mean and standard deviation. Student’s *t* test was used for statistical analysis of the patient data. Statistical calculations were performed with SPSS 13.0 (SPSS Inc., Chicago, IL, USA.). A value of *P* <0.05 was considered statistically significant.

## Results

The preoperative haemoglobin level, haematocrit level and platelet count were within the normal range in all patients. The preoperative mean haemoglobin level of Groups I and II was 13.1 and 13.0 g/dl, respectively (n.s.). The preoperative haematocrit level of Groups I and II was 40.0 and 39.4 %, respectively (n.s.). The mean tourniquet time for Groups I and II was 73 min (SD 15.5) and 74 min (SD 14.2), respectively. The average postoperative drainage volume within 48 h after surgery in Groups I and II was 724 ml (SD 166) and 767 ml (SD 153), respectively (n.s.). The calculated blood loss for Groups I and II was 1,198 ml (SD 357) and 1,272 ml (SD 349), respectively (n.s.). There were no statistically significant differences in the mean suction drainage and calculated blood loss volumes between the two groups. The mean suction drainage and calculated blood loss volumes are shown in Table 2. In each group, 36 patients received a blood transfusion. The average number of units of packed red cells transfused in Groups I and II was 1.1 and 1.2, respectively. The postoperative transfusion rates were not significantly different between the two groups.

**Table 2:** Drainage and calculated blood loss (ml)

	PCL retaining group	PCL stabilized group	*P* value
Postoperative drainage	724 ± 166	767 ± 153	n.s.
Calculated blood loss	1,198 ± 357	1,272 ± 349	n.s.

The values are given as mean and standard deviation

No significant difference was detected in the haemoglobin and haematocrit levels on the day of surgery. The haemoglobin and haematocrit levels on postoperative days 1, 3 and 5 showed no statistically significant difference between the two groups (Tables 3, 4). There were no statistically significant differences between the two groups in bleeding time, platelet count, or PTT and PT values examined just before and after surgery and on postoperative days 1, 3 and 5.

**Table 3:** Preoperative and postoperative haemoglobin values

Haemoglobin (g/dl)	PCL-retaining group	PCL-stabilized group	*P* value
Preoperative	13.1 ± 1.6	13.0 ± 1.6	n.s.
Day 0	10.8 ± 1.2	10.7 ± 1.5	n.s.
Day 1	9.7 ± 1.0	9.6 ± 1.1	n.s.
Day 3	9.7 ± 1.1	9.5 ± 0.7	n.s.
Day 5	10.9 ± 1.2	10.5 ± 0.8	n.s.

The values are given as mean and standard deviation

**Table 4:** Preoperative and postoperative haematocrit values

Haematocrit (%)	PCL-retaining group	PCL-stabilized group	*P* value
Preoperative	40.0 ± 4.6	39.4 ± 4.1	n.s.
Day 0	33.0 ± 3.7	32.4 ± 3.8	n.s.
Day 1	30.0 ± 3.0	29.5 ± 2.9	n.s.
Day 3	30.2 ± 3.2	29.4 ± 2.3	n.s.
Day 5	32.4 ± 3.3	31.5 ± 2.7	n.s.

The values are given as mean and standard deviation

## Discussion

The main finding in this study is that the PS technique does not influence the amount of postoperative blood loss or need for blood transfusion. As TKA has become a major surgery in orthopaedic practice, the influence of the operative technique on postoperative results has become a matter of debate. The amount of blood loss after TKA is usually underestimated because of the differences between the apparent and calculated blood loss [8]. Factors influencing blood loss after TKA have been attracting increasing attention in the literature. Major and old concerns about these factors have become a matter of debate and are still being investigated. For example, although the effect of tourniquet use has been a main concern in studies on perioperative blood loss since the 1990s, no consensus has yet been reached. Recent studies are still investigating the effect of tourniquet use on postoperative blood loss after TKA [16, 19]. A meta-analysis of tourniquet use in total knee arthroplasty concluded that tourniquets could decrease the measured blood loss, but could not decrease the calculated blood loss [19].

Blood transfusion, with its rare but possible complications, is another concern in terms of blood loss after TKA. According to a prospective observational study involving 18 randomly selected hospitals, a rate of blood transfusion between 12 and 87 % with a mean of approximately two units after TKA was reported [3]. Different patient populations and differences in perioperative blood loss were suggested as possible reasons for this noticeable variability. Owing to the wide range of reported blood transfusion rates after TKA, several strategies, such as autogenous blood deposition, have become popular for decreasing the blood loss volume and need for blood transfusion. The main autogenous blood deposition procedures are preoperative deposition of autogenous blood, isovolemic haemodilution, intraoperative collection of blood with cell savers and postoperative collection of blood in closed systems with continuous suction [2]. In the present study, no strategy to reduce blood loss, such as autogenous blood deposition, bipolar sealers or pharmacological agents, was used in any patient.

In addition to tourniquet application, the influences of factors such as cementing, gender, knee position and continuous passive motion on postoperative blood loss have also been investigated [1, 8, 9, 13, 14]. The main aim has been to identify factors that affect perioperative blood loss and ways to reduce this loss. Variations in results have been confusing and have led to a need for further studies. Previous studies have mostly focused on drug use, tourniquets and drainage methods. The timing of tourniquet release and the properties of tourniquets have also been subjects of research [8, 12, 14]. Stucinskas et al. compared the effect of conventional drainage and 4-h clamping drainage on postoperative blood loss and concluded that 4-h clamping drainage after TKA reduces blood loss through the drains and the need for transfusion [17]. There have also been studies on identifying the most efficient strategy for reducing postoperative haemorrhage by examining the influence of drugs such as tranexamic acid and epinephrine [6, 10, 15]. Thus, many studies, sometimes with controversial results, raise questions about the standardization of previous studies.

One of the main differences between the PS and PCR techniques is the performance of osteotomy of the distal femur. During PS TKA, an additional cut in the femoral intercondylar region results in extra blood loss from the venous sinuses of the newly appearing cancellous bone, suggesting that this technique might cause more postoperative bleeding than PCR. To the best of our knowledge, no studies have compared perioperative blood loss between the PS and PCR techniques in TKA. The lack of studies related to the effects of the PCR technique on perioperative blood loss in the literature has led to the aforementioned inadequate standardization of studies on blood loss associated with TKA.

Based on several studies, there has been no standardization of the use of either the PCR or PS technique with respect to the perioperative blood loss in TKA. Prasad et al. [13] evaluated risk factors such as gender, tourniquet time, advanced osteoarthritis and rheumatoid arthritis and concluded that gender and tourniquet time played a role in blood loss in TKA; however, the methods of the study included no information about the choice of technique. Madarevic et al. [9] compared four different methods with the variables of flexion and compression in 147 patients, but did not include information on the preference for either the PCR or PS. In another study, the timing of tourniquet release was evaluated among 11 studies involving 872 patients, but there was no mention of the choice of technique in those studies [14]. Malone et al. [10] evaluated the effect of intraarticular epinephrine lavage on blood loss following TKA in 189 patients and emphasized the limitation of his study that the results could have been affected by the fact that there were two surgeons using different techniques. The lack of standardization of the use of the PCR and PS techniques and their percentages in groups raises questions about reliability of these studies.

In the present study, although the mean haemogram and haematocrit values on postoperative days 1, 3 and 5 were slightly lower with the PS technique, there was no statistically significant difference between the two groups. The results also showed that the mean perioperative calculated blood loss and amount of drainage with the PS technique were slightly higher, but this difference was statistically negligible and insignificant. Comparison of the postoperative blood transfusion rate revealed no statistically significant differences between the groups. Thus, preference for the PS technique did not affect the need for postoperative transfusion. All of these findings of the present study are clinically useful for surgeons in terms of choosing either the PCR or PS technique. The main clinical relevance of this study is that the difference in blood loss between the PCR and PS techniques does not need to be considered by surgeons when performing TKA. Furthermore, a higher amount of drainage could be seen in some patients after TKA; in such cases, surgeons must attempt to identify the underlying problem, such as a bleeding disorder, surgical complication or metabolic disorder. The surgeon and anaesthesiologist should not expect differences in blood loss between the PS and PCR techniques.

There are some limitations to this study. First, this study was not a double-blind study; the evaluator was not blinded during the evaluation process. The results are only those of cemented TKA. It was reported that blood loss in cementless TKA is significantly higher and that cementation decreases blood loss [2]. The study needs to be repeated with cementless TKA to reach a more generalized conclusion. In the same study, cementation did not decrease the average blood loss in patients with rheumatoid arthritis, and it was shown that these patients had different blood loss patterns from those of patients from primary osteoarthritis [2]. The patients in the present study had primary knee osteoarthritis; cases of knee osteoarthritis secondary to other disorders such as rheumatological disorders were not evaluated. In addition, computer-assisted surgery has been shown to cause less bleeding than conventional techniques [5, 7]. Therefore, the use of an additional system such as computer-assisted surgery may also influence the results. Finally, this study did not evaluate the results of TKA without a tourniquet or with the use of a tourniquet at different pressures.

## Conclusion

Compared with the PCR technique, use of the PS technique in TKA does not appear to influence the amount of postoperative blood loss or the need for postoperative blood transfusion in general. The difference in blood loss between the two techniques does not need to be considered by surgeons when performing TKA.
